# Mediastinal malignant rhabdoid tumor in an infant: A rare case report

**DOI:** 10.1016/j.radcr.2024.02.070

**Published:** 2024-03-21

**Authors:** Elham Zarei, Omid Alemohamad, Zahra Rahimi, Ali Manafi Anari, Behzad Haghighi Aski, Nafise Mortazavi, Maryam Sakhaei, Golnaz Gharebaghi, Amir Ghadipasha

**Affiliations:** a Hazrat Ali Asghar Children Hospital, Iran University of Medical Sciences, Tehran, Iran; b School of Medicine, Iran University of Medical Sciences, Tehran, Iran; c Firoozabadi Clinical Research Development Unit (FACRDU), Iran University of Medical Sciences, Tehran, Iran

**Keywords:** Rhabdoid tumor, Mediastinal neoplasms, Infant, Case reports, Rare diseases

## Abstract

Mediastinal malignant rhabdoid tumor (MRT) is an exceedingly rare and aggressive neoplasm, particularly uncommon in infants. We present the case of a previously healthy 7-month-old male infant with mediastinal MRT. The patient initially presented with left eyelid ptosis and was otherwise asymptomatic. Initial investigations, including brain MRI, yielded unremarkable results, and the infant was discharged with vitamin B supplements. However, he was readmitted a week later with prolonged fever, poor feeding, diarrhea, and respiratory distress. Despite an initial diagnosis of bronchiolitis/viral respiratory tract infection, the patient's condition rapidly deteriorated. Subsequent evaluation revealed mediastinal MRT as the underlying cause. This case underscores the diagnostic challenges associated with mediastinal MRT in infants and highlights the importance of considering rare neoplastic etiologies in atypical clinical presentations.

## Introduction

Malignant rhabdoid tumor (MRT) is a rare and highly aggressive neoplasm that predominantly affects young children, often presenting at an advanced stage [1,2]. The nomenclature encompasses three recognized forms of MRT, reflecting their anatomic localization: malignant rhabdoid tumor of the kidney (MRTK), atypical teratoid rhabdoid tumor involving the central nervous system, and extrarenal extracranial rhabdoid tumor (EERT) [3]. In this report, we present the case of a 7-month-old male infant diagnosed with mediastinal MRT. The patient's clinical presentation and diagnostic pathway are detailed to provide insights into the challenges associated with this rare condition.

## Case report

A previously healthy 7-month-old male infant was admitted due to left eyelid ptosis beginning 2 days ago. He had no recent infection, vomiting, seizure, or head trauma history. He was the result of a full-term pregnancy with a birth weight of 2800 grams. His prenatal history was unremarkable. On physical examination, he appeared well, and vital signs were within the normal range. He was conscious, and developmentally normal. His current weight, height, and head circumference were within normal ranges. There was hardly noticeable ptosis on the left side with concordant pupillary miosis. Extraocular muscles and other cranial nerves examination appeared normal bilaterally. Corneal reflexes to light are normal although the left eye was miotic. Deep tendon reflexes on all 4 limbs appear 2+, and muscle tone in extremities was normal.

**Fig. 1:**
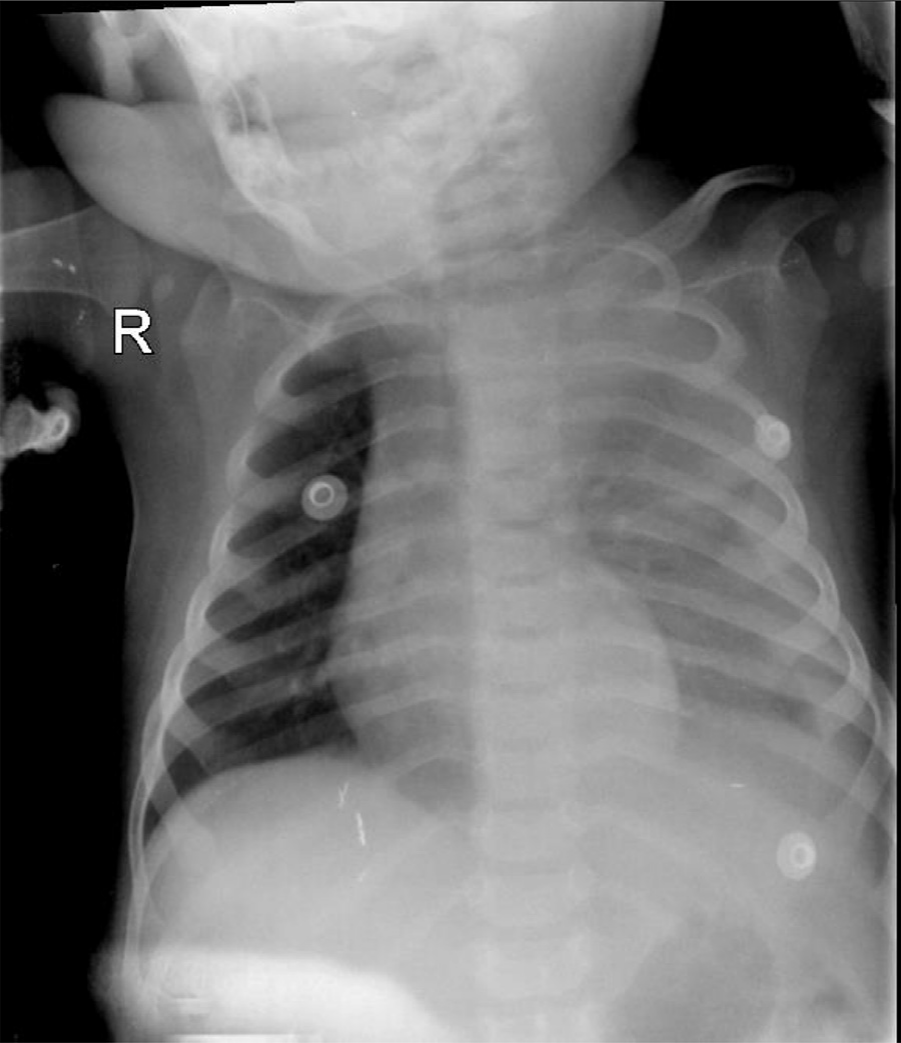
CXR show opacity of upper zone of Lt lung and ground glass opacity of other parts of Lt lung along with adjacent pleural effusion and mediastinal shift to Rt side.

MRI of the brain is performed to rule out intracranial pathology and was reported normal. Given the acceptable general condition of the infant, his parents are educated about the red flags and asked to return for a follow-up appointment a month later. finally, vitamin B supplements are prescribed, and the infant is discharged. About a week later, the patient returned to the emergency department complaining of prolonged fever, poor feeding, diarrhea, and respiratory distress. He was febrile (38°C), dehydrated, tachypneic with normal blood pressure. On pulmonary auscultation, there was mild, generalized, end-expiratory wheezing heard in both lung fields. He was admitted to the general ward with the initial diagnosis of bronchiolitis/viral respiratory tract infection. After a partial response to treatment, the patient's clinical condition suddenly deteriorated with respiratory distress and progressive deterioration in oxygen saturation. The patient is admitted to the intensive care ward, where high-flow oxygen is initiated through a nasal cannula and broad-spectrum antibiotics were administered and a CXR was taken (Fig. 1).

**Fig. 2:**
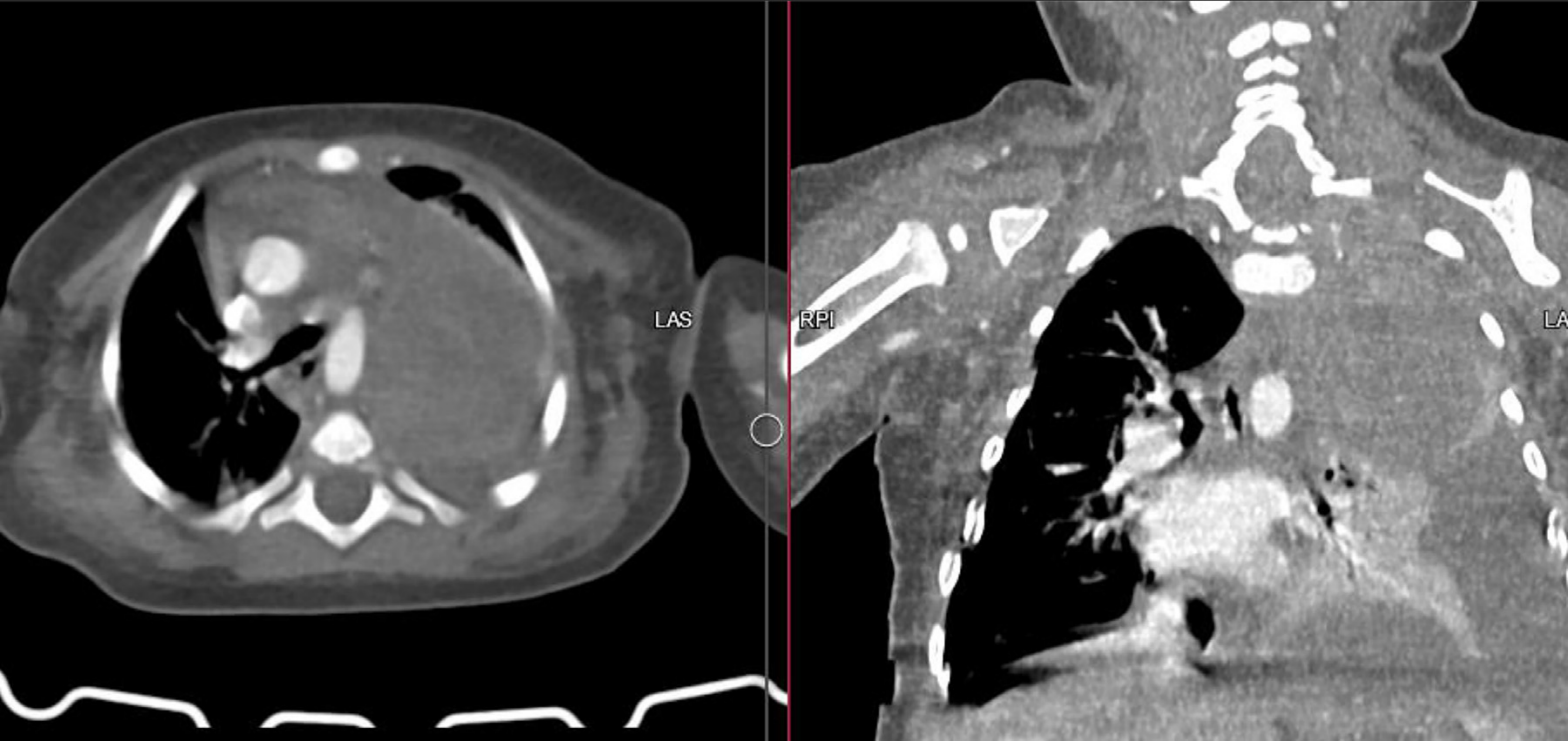
Contrast enhanced Ct SCAN show an ill-defined hypodense mass that occupies nearly entire of upper zone of Lt hemithorax that causes collapse of nearly entire of Lt lung and mediastinal shift to Rt side along with adjacent moderate pleural effusion.

Regarding to CXR findings, chest ultrasound was performed, which showed a moderate pleural effusion containing diffuse internal echo with a heterogeneous mass in the upper part of the Lt hemithorax. Ultrasound-guided pleural effusion aspiration is performed that the bloody, exudative fluid with a WBC count of 20000/mm (90% PMNs), RBC count of 1640000/mm, Glucose of 88 mg/dL and protein of 4.3 g/dl supports a non-infectious, malignant involvement. After the patient was stable, a CT scan with IV contrast of the chest is performed which confirmed the ultrasound findings (Fig. 2).

**Fig. 3:**
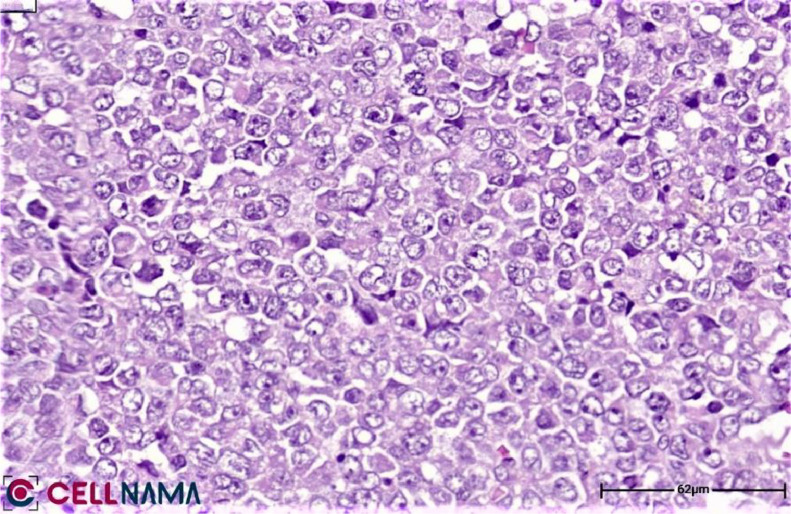
The tumor fragment consists of sheets of cohesive, small round to polygonal cells with rhabdoid morphology (spherical nuclei and prominent nucleolus) (H&E Stain, original magnification  × 400).

Because of previous Horner's syndrome, the current thoracic mass was suspected of being neuroblastoma, additional evaluations were performed. About 24-hour urinary catecholamines are measured and reported as normal. Bone marrow aspiration and biopsy were performed, showing no evidence of primary or metastatic involvement. Therefore, neuroblastoma is excluded. An ultrasound-guided core needle biopsy is performed for further evaluation. The pathology report was Rhabdoid tumor (Fig. 3).

**Fig. 4:**
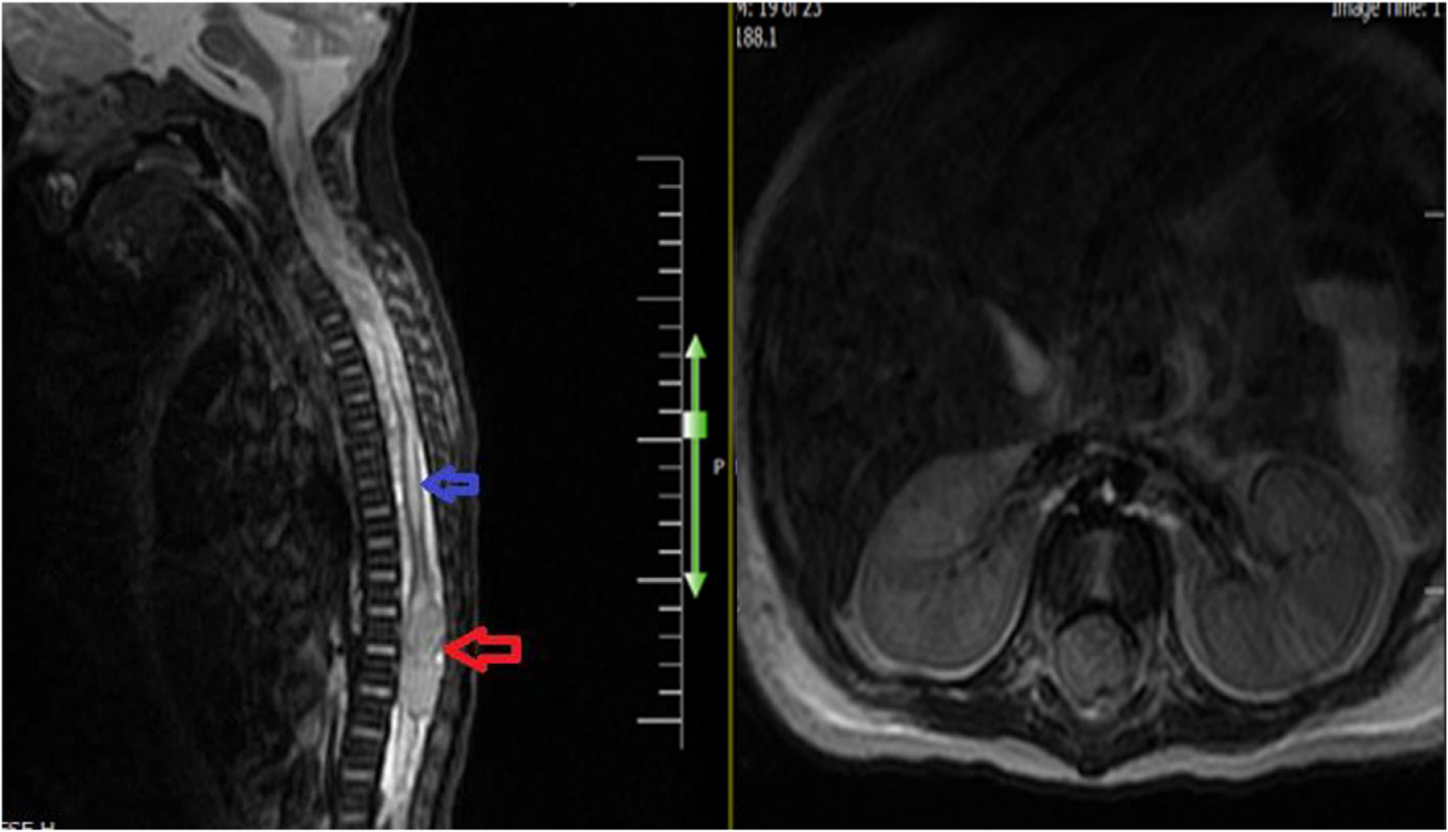
The sagittal T2 w image showed a large epidural mass (Red arrow) in the lumbar region (L1-L3) along with proximal syringomyelia (Blue arrow) .

The positive staining for CD99, cytokeratins, including PAN keratins, EMA, and low MW keratins coupled with the loss of INI-1/BAF47 stain reaction best fitted to the immunophenotype of the rhabdoid tumor. IHC for brachyury was negative, ruling out the diagnosis of chordoma/dedifferentiated chordoma. Pleuropulmonary blastoma and embryonal rhabdomyosarcoma were not favored. Negative staining for myogenin and SMA further suggested that the tumor was not a rhabdomyosarcoma. Negative desmin staining of the tumor for WT1 was not supportive of the desmoplastic SRC tumor. Chemotherapy was started and the patient underwent surgery for total / subtotal mass resection. The surgical team reported an extra-parenchymal mass with the possible origin of pleura in the upper zone of the left hemithorax. .The tumor was partially resected due to its proximity to the left subclavian artery and chest tubes were placed for hemithorax. After surgery, the patient's general condition, specifically his respiratory distress, improved significantly, as he no longer required supplemental oxygen. Two days later, however, he developed hypertension, bradycardia, and a sunset eye. On an emergent brain CT scan, there was severe communicating hydrocephalus. The patient was operated on emergency and a ventriculoperitoneal shunt was placed. A CSF sample was taken during surgery and sent for laboratory evaluation that was unremarkable. For further evaluation, an brain and spinal MRI without IV contrast was performed (Fig. 4).

A large epidural mass was seen in the lumbar region (L1-L3) along with proximal syriongomeylia. After two days, because the movement of the lower extremities was gradually decreasing, the neurosurgical team candidate the patient for spinal decompression surgery. The patient underwent L1-L4 laminectomy; a vascularized lobulated epidural mass was seen in lumbar region. The tumor involved nerve roots. It was resected as much as grossly visible, and the specimen was sent for pathologic examination. After his 5th chemotherapy session, the patient developed vomiting, diarrhea, and fever and was admitted for further workups and treatment. Routine lab tests, Blood and Catheter cultures, and nasopharyngeal sample PCR for Adenovirus and SARS-COV-2 were taken and conservative management alongside Empirical antibiotics was initiated. The blood culture results were positive for Klebsiella pneumonia and despite directed antibiotic treatment, the patient's general condition gradually worsened as he developed multiple organ failure. Unfortunately, he passed away due to complications from septic shock.

## Discussion

MRT is a rare, aggressive neoplasm predominantly occur in young children [1,2]. Although most of these tumors arise from kidney and CNS,recent studies reported that MRT occurred in nearly every anatomical site [1,3,4]. The incidence of MRT is estimated at 0.6 per million [5]. Mediastinal EERT in pediatric population is very rare and to the best of our knowledge, only 29 cases have been reported in the literature [[2], [3], [4],[6], [7], [8], [9], [10], [11], [12], [13]]. The details of these patients are not available in most articles due to being reported as single cases in MRT case series. The age at presentation for mediastinal EERT varied from newborn [11] to 13 years old [2,8].

Regardless of the anatomic origin, MRT is genetically characterized by mutation of the tumor suppressor gene SMARCB1/INI1 on chromosome 22q [3]. In the reviewed articles, some studies have shown 100% [6] and 98.11% [3] of the MRT patients had loss of INI1 staining whereas Hoot et al. reported that up to 20% MRTs have no alterations in the INI1 gene at the DNA or RNA level, and that loss or mutation of the SMARCA4 (BRG1) gene is a much less frequent molecular alteration characteristic of MRT [12]. Also, some studies have shown that epithelioid sarcoma, renal medullary carcinoma may loss of INI1 expression [6]. The diagnosis of MRT still requires tumor tissue microscopic morphology combined with immunohistochemical results [6]. Rhabdoid tumors typically show large vesicular nuclei, prominent nucleoli and, focally, cells demonstrate the characteristic eosinophilic cytoplasmic inclusions [7].

The clinical manifestations of MRT varies depending on tumor location. Mediastinal EERT usually presents with chest pain, dyspnea, cough, and respiratory distress, which are nonspecific but are likely due to the mass effect of the tumor [8,11]. Siqi et. Al reported 2 cases of mediastinal MRT with airway compression symptoms [6]. Similar to our case, Gururangan et. Al reported a 13 year old boy with mediastinal MRT presenting with chest pain and Horner syndrome [8]. On CT and MRI all EERT tumors were predominantly solid, lobulated, heterogeneous enhancing masses with mass effect on adjacent structure or infiltrative feature [7,11].

Age at diagnosis was a highly significant prognostic factor in a review of 142 patients who had MRTK. The 4-year survival rate for the youngest group of patients (0-5 months) was 8.8% compared with 41% for patients who were diagnosed at age ≥24 months [14]. Another study confirmed that patient age only significantly influenced overall survival, with those younger than 1 year of age, having a significantly worse outcome, with a 4-year OS of 20.1% and the 1‐year survival was only 31%[4]. In a 2022 study The 1-year overall survival (OS) of 71.4% at the age of ≥24 months was significantly higher than the 1-year OS (11.1%) within age 24 months [6].

Risk of death was also related with gender in metastatic patients with male patients having worse prognosis [4]. Also higher tumor stage and presence of a CNS lesion were both shown to be predictive factors of a poor survival rate [14]. In a single-institution series of 14 cases of extracranial MRT diagnosed over a 20-year period, the median time to progression was only 2 months [1]. Tumor progression or recurrence occurred in 75.47% [3], 67% [4] of MRT cases with a median time to progression of 76 days, 5.0 months after diagnosis respectively. Given the rarity of MRT, standardized treatment protocols have not yet been popularized worldwide. Generally, treatment protocols are based on a multimodal approach, combining surgery, chemotherapy, and radiotherapy [3]. Despite many attempts to improve various treatment regimens, MRT is still described as lethal.

Previous studies suggested that radical surgery seemed to benefit the prognosis in MRTK [1,3]. Analysis showed that use of radiotherapy was significantly associated with improved survival [3,5,9]. In 2010, Sultan et al. confirmed that age, stage, and radiotherapy, but not primary tumor site, affected the outcome of MRT patients [5]. Their univariate and multivariate analyses showed that age at diagnosis (> 2 years), localized stage, and use of radiotherapy were significantly associated with improved survival [5]. However, in these studies, radiotherapy was often given to those with a higher clinical stage and to groups with older patients and It might be possible that radiation contributed to the improved survival of MRT in older patients who were irradiated to higher doses than younger patients. Therefore, the role of radiotherapy still needs to be analyzed by randomized controlled trials in the future.

## Conclusion

Extrarenal -extracranial MRT is a rare,highly aggressive childhood tumor associated with poor prognosis. Although the imaging feature of these tumors are nonspecific and biopsy is necessary for confirmation the diagnosis,it is necessary to keep in mind MRT in the differential diagnosis of tumors with aggressive feature on imaging in children under 2 years of age.

## Patient consent

Written informed consent for the publication of this case report was obtained from the patient's parent.
